# Removal of Cadmium and Lead from Contaminated Soils Using Sophorolipids from Fermentation Culture of *Starmerella bombicola* CGMCC 1576 Fermentation

**DOI:** 10.3390/ijerph15112334

**Published:** 2018-10-23

**Authors:** Xiaoyu Qi, Xiaoming Xu, Chuanqing Zhong, Tianyi Jiang, Wei Wei, Xin Song

**Affiliations:** 1 School of Municipal and Environmental Engineering, Shandong Jianzhu University, Jinan 250101, China; qixiaoyu2006@sdjzu.edu.cn (X.Q.); xuxiaoming@sdjzu.edu.cn (X.X.); zhongchuanqing@sdjzu.edu.cn (C.Z.); jiangtianyi@sdjzu.edu.cn (T.J.); 2 State Key Laboratory of Microbial Technology, Shandong University, Bin Hai Road 72, Qingdao 266237, China; 3 Environmental Monitoring Center of Shandong Province, Jinan 250101, China; weiweilinyl@126.com

**Keywords:** sophorolipids, cadmium, lead, contaminated soil, bioremediation, *Starmerella bombicola* CGMCC 1576

## Abstract

Soil contaminated with Cd and Pb has caused sharp decrease of cultivatable soil and has been attracting increasing attention. Biosurfactants are efficient in solving the problem. However, little information is available about the influence of sophorolipids (SLs) on the remediation of Cd- or Pb-contaminated soil. The sophorolipids produced by *Starmerella bombicola* CGMCC 1576 were used to study the effects of Cd and Pb removal in batch soil washing from artificially contaminated soil. The removal efficiency of crude total SLs was better than both distilled water and synthetic surfactants. Furthermore, 83.6% of Cd and 44.8% of Pb were removed by 8% crude acidic SLs. Acidic SLs with high water solubility were more effective than lactonic SLs in enhancing remediation of heavy metal-contaminated soils. The complexation of Cd with the free carboxyl group of the acidic SLs was observed by Fourier-transform infrared spectroscopy study, and this complexation was effective in heavy metal removal from the soil. The fermentation broth of *S. bombicola*, without further preparation, removed 95% of Cd and 52% of Pb. These results suggested that SLs produced by *S. bombicola* could function as potential bioremediation agents for heavy metal-contaminated soil.

## 1. Introduction

With the development of mining, the use of pesticides and batteries, and discharge of industrial waste, urban and agricultural soils are often contaminated with heavy metals. Excess heavy metals in soils could pollute the environment and potentially damage human health through accumulation in the food chain. Cadmium (Cd) and lead (Pb) are listed as prior heavy metal pollutants for their effects on the brain, kidney, and liver [1]. Moreover, a great number of heavy metal-contaminated sites have been reported both in North America and China [2,3]. Therefore, the effective control and remediation of heavy metal-polluted soils has attracted increasing attention.

Many chemical, physical, and biological methods have been suggested to solve the problem of heavy metal contamination, in which soil washing is one of the most effective treatments [4,5]. Soil washing is an ex (or in) situ technology using a water-based process and relies on traditional chemical and physical extraction and separation processes for removing a broad range of organic and inorganic contaminants from soils [6]. A range of washing agents, including inorganic acids, organic acids, chelating agents such as ethylene diamine tetraacetic acid (EDTA), synthetic surfactants, and biosurfactants, are used for recovering heavy metals from soils [7,8]. In these washing agents, synthetic surfactants are effective chemical agents in the removal of heavy metals from contaminated soil [9,10]. However, synthetic surfactants or chelating agents used in soil remediation might become secondary contaminants because of their toxicity and resistance to biodegradation.

Recent studies have shown that biosurfactants are more efficient and environmentally friendly than synthetic surfactants. Biosurfactants, such as rhamnolipids, surfactin, saponin, and sophorolipids (SLs), have been employed to remove lead (Pb), cadmium (Cd), nickel (Ni), copper (Cu), and zinc (Zn) from contaminated soils [11,12,13,14]. Biosurfactant concentration, pH value, and soil washing method (batch-stirred reactor or column; single or series washing) could affect the removal efficiency of Cd and Pb. Juwarkar et al. [15] found that more than 92% of Cd and 88% of Pb were removed under column-operated continuous mode with 0.1% di-rhamnolipid at pH 8.0 (adjusted with NaOH). Among the different types of biosurfactants used for bioremediation of heavy metal-contaminated soil, sophorolipids are one of the most promising biosurfactants because of their good surface activity, biodegradability, biocompatibility, low toxicity, high productivity, and production from renewable materials [16,17,18].

Sophorolipid (SL) is a type of extracellular glycolipid biosurfactant produced by some species of nonpathogenic yeast, such as *Rhodotorula bogoriensis*, *Candida bombicola*, *Candida batistae*, etc. The yields of SLs were the highest among all the biosurfactants. They can reach up to 400 g/L and have been used for commercial production and applications [19]. On the other hand, cheap and fermentative feedstocks have been used to reduce the production cost of SLs, which makes large-scale applications of sophorolipids be feasible [20]. Heavy metal removal efficiency from oil-contaminated soil and contaminated canal sediment by sophorolipid (SL), rhamnolipid and surfactin were compared [11,21]. The results showed that SL (with HCl) and rhamolipid (with NaOH) performed well in heavy metal removal and SLs showed better Zn and Cu removal efficiency in some oil-contaminated soil, therefore, applying SLs in the remediation of contaminated soil was feasible and advantageous. SLs can be classified as acidic (anionic) or lactonic (nonionic); however, few studies regarding the application of SLs, especially acidic SLs, in the remediation of Cd- or Pb-contaminated soil were available.

This study investigated the efficiency of different types of SLs produced by *Starmerella bombicola* CGMCC 1576 in the bioremediation of Cd- and Pb-contaminated soil samples. Pb and Cd removal by crude total SLs and synthetic surfactants was studied. Removal efficiency of Cd and Pb was evaluated using crude acidic and lactonic SL solutions as washing agents. The fermentation broth of *S. bombicola* was also applied for direct removal of Cd and Pb from soils. Fourier-transform infrared (FT-IR) spectroscopy was employed to study the mechanism of heavy metal removal by acidic SLs as washing agents in the article.

## 2. Materials and Methods

### 2.1. Chemicals

All the chemicals were of analytical grade. Cadmium sulfate (3CdSO_4_·8H_2_O, AR grade) and lead acetate ((CH_3_COO)_2_Pb·3H_2_O, AR grade) were purchased from Guangdong Xilong Chemical Factory Co., Ltd., Shantou, China. Sodium dodecyl sulfate (SDS) was obtained from AMRESCO Inc. (Solon, OH, USA). Other chemicals and reagents were purchased from Sinopharm Chemical Reagent Co., Ltd., and Shanghai Tingxin Chemical Factory Co., Ltd., Shanghai, China.

### 2.2. Soil Sample Collection and Experimental Design

A surface clay soil sample (0–20 cm) was collected from the campus of Shandong Jianzhu University, Jinan, China and confirmed to be free of Cd or Pb contamination with organic carbon content of 0.66% and pH 6.93. Soil was artificially contaminated in our laboratory with solutions of 456 mg/L 3CdSO_4_·8H_2_O (soil sample A) or 732 mg/L (CH_3_COO)_2_Pb·3H_2_O (soil sample B) according to the risk control standard of Cd and Pb for soil contamination of agricultural land [22]. The two contaminated soil samples were shaken in a shaker for 5 days at room temperature, and then centrifuged at 6000 rpm for 15 min to remove the unadsorbed metals. The supernatant was discarded, and the contaminated soil was air dried over half a year and sieved through 200-mesh sieve [15]. The final concentrations of Cd and Pb were 142 and 265 mg/kg dry soil, respectively.

Soil samples A and B were batch washed with different surfactant solutions to remove Cd or Pb. Three types of crude SLs (total SLs, lactonic SLs, and acidic SLs) and two types of synthetic surfactant (SDS and Tween-80) were used as batch soil washing agents. Six concentrations (*w*/*v* %; deionized water as control and recorded as 0%) were prepared for each surfactant to evaluate the removal efficiency of Cd or Pb. Fermentation broth of *S. bombicola* as batch washing agent was diluted to five different concentrations (*v*/*v* %) to evaluate their performance on Cd or Pb removal from contaminated soil.

### 2.3. Crude SLs Preparation and Analysis

The sophorolipids applied in this study were produced by *Starmerella bombicola* CGMCC 1576 (formerly *Wickerhamiella domercqiae* var. *sophorolipid* CGMCC 1576) [23]. The *S. bombicola* strain was cultivated in yeast extract peptone dextrose medium as seed medium [24]. The fermentation medium contained the following compositions (grams per liter): glucose, 80.0; yeast extract 3.0; KH_2_PO_4_, 1.0; Na_2_HPO_4_·12H_2_O, 1.0; MgSO_4_·7H_2_O, 0.5; rapeseed oil, 60.0. The strain was cultivated in seed medium on a rotatory shaker at 180 rpm, 30 °C for 24 h, and then 2% (*v*/*v*) of the culture was transferred to fermentation medium and cultivated for 7 days at 180 rpm, 30 °C.

Crude total SLs, lactonic SLs, and acidic SLs were prepared as follows [25]: Two volumes of ethanol were added to the fermentation broth and then centrifuged at 10,000 rpm for 10 min and evaporated by vacuum evaporation; the residue from the previous step was washed twice with n-hexane and evaporated again by vacuum evaporation at 50 °C to obtain total SLs (mixture of acidic and lactonic SLs). For preparation of lactonic and acidic SLs, two volumes of ethyl acetate were added to the fermentation broth; the lactonic SLs were in the organic phase and the acidic SLs were in the aqueous phase. Then the organic phase was concentrated by vacuum evaporation to remove ethyl acetate, the collected residue was washed twice with n-hexane to remove residual rapeseed oil and evaporated by vacuum evaporation to remove n-hexane at 50 °C to obtain the lactonic SLs. Two volumes of alcohol were added to the aqueous phase containing acidic SLs and centrifuged at 10,000 rpm for 10 min, after which the supernatant was concentrated by vacuum evaporation at 50 °C; the residue from the previous step was washed twice with n-hexane and evaporated again by vacuum evaporation to obtain the acidic SLs.

The amounts of total, acidic, and lactonic SLs were assayed by the anthrone method [25].

### 2.4. Batch Soil Washing Experiments

The soil washing process with surfactants was performed in batch experiments. Soil (1 g) was washed with 10 mL of deionized water or 10 mL of deionized water containing surfactant (or sophorolipid biosurfactant) of different concentrations. A series of washings were performed by washing the soil for 24 h in glass vials on a rotatory shaker at 150 rpm, room temperature; the supernatant was collected by centrifugation (6000 rpm for 15 min) and then fresh surfactant solution was added again. The procedure was repeated four times. The supernatants were stored at 4 °C for further analysis.

### 2.5. Pb and Cd Testing

The supernatant was digested by microwave-assisted digestion (CEM Corporation, Matthews, NC, USA) with HNO_3_ and HCl [26]. The metal concentrations of digestion solutions were then analyzed using inductively coupled plasma atomic emission spectrophotometer (ICP-AES, iCAP6300, Thermo Elemental Corporation, Waltham, MA, USA). The percentage metal removal was determined based on the initial metal content in the soil.

### 2.6. Data Analysis

Data was analyzed using IBM SPSS Statistics 22.0 (IBM Corp, Armonk, NY, USA). Analysis of variance was used to assess statistical significance of treatments, followed by a multiple comparison test of the means (Bonferroni test and Tukey’s honest significant difference test); the significance level was assessed at 0.05 and error bars were plotted based on the standard deviation.

### 2.7. FT-IR Analysis

FT-IR spectrometer (Nicolet iS10, Thermo Fisher Scientific, Waltham, MA, USA) was used to investigate the changes in the chemical groups of 8% crude acidic sophorolipids interacted with CdCl_2_ in the solution by the KBr pellet method [25]. The analysis conditions were measured in the range of 4000–350 cm^−1^ at a 0.4 cm^−1^ resolution.

## 3. Results and Discussion

### 3.1. Comparison of Pb and Cd Removal by Crude Total Sophorolipids and Synthetic Surfactants at Different Concentrations

**Figure 1:**
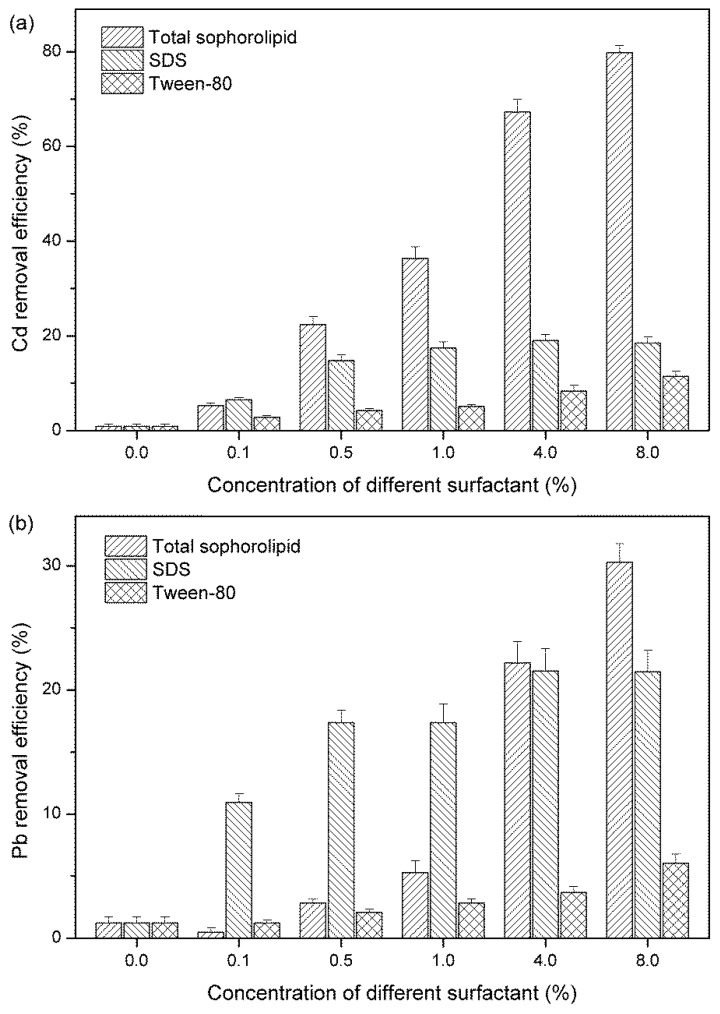
Removal efficiency of Cd (**a**) and Pb (**b**) after batch washing with crude total SLs and synthetic surfactants.

Biosurfactants have many advantages over chemical surfactants, such as biodegradability, compatibility with the environment, and low toxicity. In this study, we compared the heavy metal removal efficiency of crude total SLs produced by *S. bombicola* with that of two synthetic surfactants (Tween 80 and SDS). The removal efficiency of Cd by different washing solutions was presented in Figure 1a. The surfactant concentration influenced the Cd removal efficiency. Apart from 0.1% surfactant, total SLs had much higher removal efficiencies than those of Tween 80 or SDS at concentrations of 0.5%~8%. However, the removal rates of Pb showed different trends (Figure 1b). SDS showed the best results among the three different types of surfactants at low concentrations (0.1%, 0.5%, and 1%). At 8%, total SLs exhibited higher efficiency than SDS. The removal efficiency of Pb by Tween 80 was significantly lower than total SLs and SDS when the concentration was higher than 0.5%. Total SLs at concentrations of 0.1–8% removed 5.28–79.75% of Cd and 0.47–30.28% of Pb.

SLs showed superior performance than synthetic surfactants in the remediation of heavy metal-contaminated soils. The results were similar to previous reports about other biosurfactants, such as di-rhamnolipid and sponin. The removal performance for Cd and Pb was strongly affected by the concentrations of biosurfactant and the initial metal, soil, and reactor type [15,27]. Similar to other types of biosurfactant (e.g., rhamnolipid, aescin, and saponin), the removal efficiency of Pb by SLs was lower than that of Cd [15,27,28]. The greater sorption capacity of soils for Pb compared with Cd could help explain the results [29].

Surfactants could remove heavy metals by ion exchange, precipitation dissolution, and counterion association [21,30]. The anionic surfactant SDS removed Cd and Pb from the contaminated soils more effectively than nonionic surfactant Tween 80 (*p* < 0.05). The crude total SLs enhanced heavy metal desorption probably because of the formation of ionic bonds or surfactant micelles.

The concentration of biosurfactant is a critical factor influencing heavy metals removal efficiency. Torrens et al. [31] reported that the removal of soil-bound Cd depended on the amount of rhamnolipid in the solution phase. The mobilization of metals or organic contaminants from soils and sediments increased when rhamnolipids formed micelles at concentrations above the critical micelle concentration (CMC) [32]. Similar to rhamnolipids, the removal efficiencies of Cd or Pb increased with the increasing concentration of SLs in the soil washing solutions.

### 3.2. Removal Efficiency of Crude Acidic and Lactonic SLs on Cd and Pb

**Figure 2:**
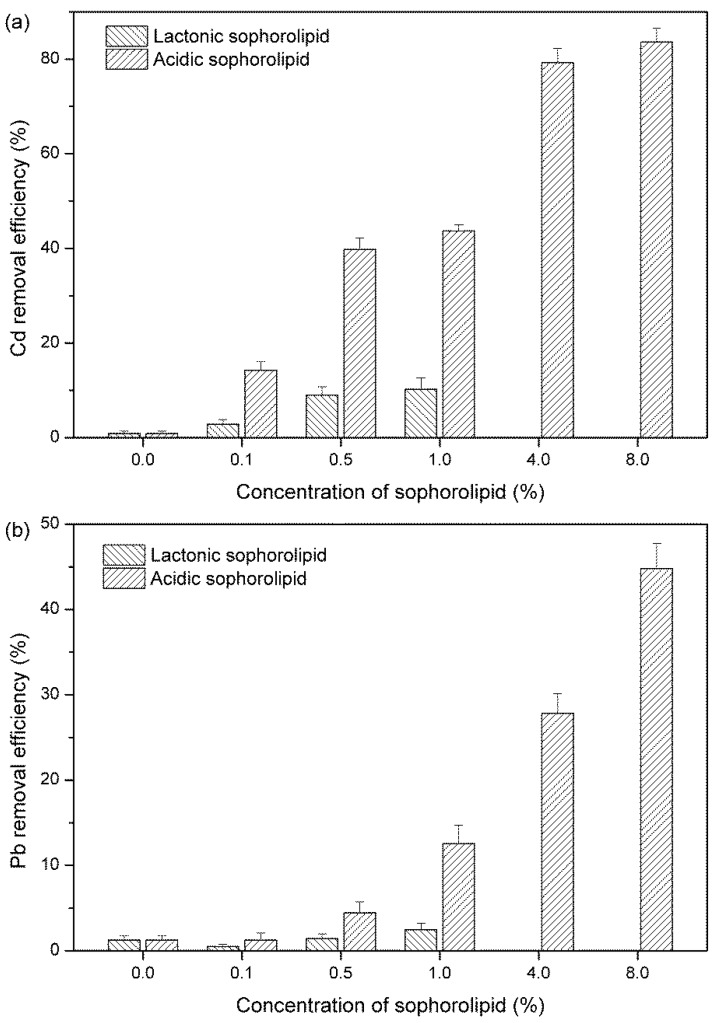
Removal efficiency of Cd (**a**) and Pb (**b**) by acidic and lactonic SLs.

Total SLs comprised lactonic and acidic SLs. With their different chemical structures, lactonic and acidic SLs should have different elution results. The removal efficiencies of Cd and Pb by crude acidic and lactonic SLs at different concentrations were shown in Figure 2. Washing with deionized water (control experiment) enabled removal efficiencies of only 0.88% for Cd and 1.23% for Pb. With addition of acidic or lactonic SLs (1%, *w*/*v*), Cd removal was enhanced to 43.7 and 10.2%, respectively. For Pb, the removal efficiency by 1% acidic SLs was nearly 13%. However, a low concentration (0.5%) of lactonic SLs did not show significant improvement in Pb removal compared with the control (*p* > 0.05), similar to a previous report [33]. Because the solubility of lactonic SLs in water was very low, the removal efficiency of crude lactonic SLs for heavy metals at concentrations of 4% and 8% were not studied. When SLs concentration increased to 4%, crude acidic SLs showed superior efficiency of Cd and Pb removal (79% and 28%, respectively). The maximum Cd removal reached over 83% when the acidic SLs concentration was 8%. At the same concentration, Pb removal rate was 45%, which was 3.6-fold higher than that treated by 1% acidic SLs. Chang et al. [34] summarized that anionic surfactants could enhance metal removal from soil through the mechanisms of counterion exchange at micelle surfaces above the CMC, while nonionic surfactants might be adsorbed onto soil or have no effects on metal removal. Our results suggested that, as a type of anionic surfactant, acidic SLs showed better removal efficiency of Cd and Pb from soils than nonionic lactonic SLs (*p* < 0.05).

According to Miller [35], heavy metals desorption from solid phases by adding biosurfactant can be promoted in two ways. The first is through complexation of the free form of the metal residing in solution. The second is that under conditions of reduced interfacial tension, biosurfactants in contact with metals will accumulate at the solid-solution interface. Anionic surfactants were able to enhance heavy metal removal from soil in previous reports [36], the probable reason for enhanced heavy metal removal was that anionic surfactants increased adsorption of metals to the surfaces of surfactants by forming metal-surfactant complexes [37]. The anionic surfactants are negatively charged, so when the molecule encounters a metal cation, such as Cd^2+^ or Zn^2+^ that carries a positive charge, an ionic bond is formed [38]. The micelles help remove the metals from soil surfaces, reduce interfacial tension, and transport them into solution; the metals then become easier to remove by flushing [39,40]. Acidic SLs carry a negatively charged head group (COO−) and are more water soluble than lactonic SLs, so they showed higher removal efficiency of Cd and Pb from contaminated soil. The removal rate of heavy metals was positively related to the concentration of acidic SLs.

### 3.3. Effect of Fermentation Broth of S. Bombicola on Heavy Metal Removal

**Figure 3:**
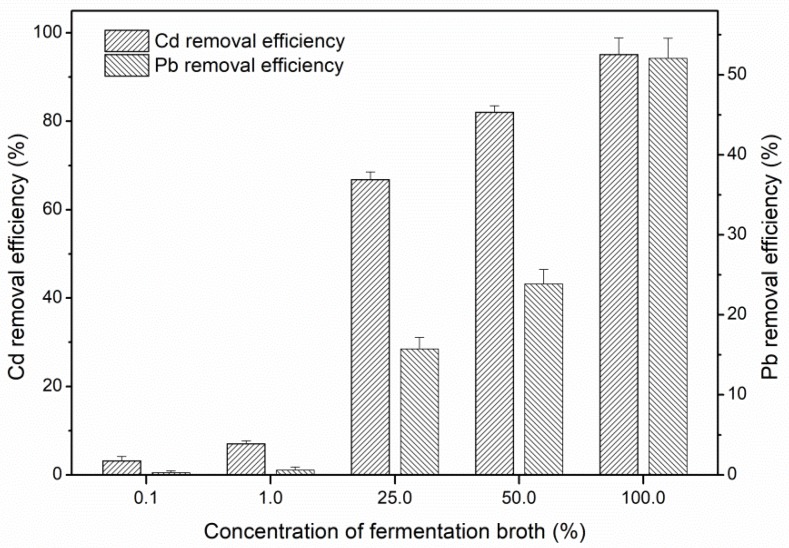
Removal of heavy metals from contaminated soil at different concentrations (*v*/*v* %) of *S. bombicola* fermentation broth.

For practical application of SLs for heavy metal removal from soil, heavy metal removal efficiency of fermentation broth of *S. bombicola* (pH 3.1) was investigated. The fermentation broths of *S. bombicola* with various concentrations were applied. Increased concentrations of fermentation broth could enhance Cd and Pb removal (Figure 3). The fermentation broth with yeast extract as nitrogen source was more efficient for Cd and Pb removal than crude SLs. When the concentration of fermentation broth of *S. bombicola* was lower than 1%, Cd and Pb could not be effectively removed. However, 67% of Cd was removed at a broth concentration of 25%. With the addition of 50% fermentation broth solution, 82% of Cd and 24% of Pb were removed from contaminated soil. Maximum removal rate of Cd and Pb after four rounds of washing reached 95 and 52%, respectively, when soil samples were washed with undiluted fermentation broth (100%).

It was reported that the pH value of the washing solution plays a significant role in removing heavy metals from soils [7]. In this study, we compared the Cd and Pb removal efficiency by different types of SLs washing solution under their natural pH. Washing with dilute hydrochloric acid (pH 2.5), the Cd and Pb removal efficiency was 2.64% and 4.3%, respectively. However, the 8% crude total or acidic SLs solution with the same nature pH 2.5 could remove about 80% of Cd or 30% of Pb, respectively (Figure 1 and Figure 2). The removal efficiency of Cd and Pb by 4% crude acidic SLs solution (nature pH 2.7) was significantly higher than by the same concentration and pH of crude total SLs washing solution with it (Figure 1 and 2). The highest removal efficiency of Cd and Pb was using the fermentation broth as washing fluid under nature pH 3.1 (Figure 3). These results demonstrated that the efficiency in removing heavy metals from soils was probably not attributed to low pH of biosurfactant solutions. It is hypothesized that the higher removal efficiency of the fermentation broth could be due to the more anion groups of the acidic SLs exposure under proper conditions such as pH value and biosurfactants concentration in the fermentation. This provides more binding sites for metal cations on the biosurfactant, thus increasing metal solubility [15,40]. Therefore, fermentation broth of *S. bombicola* was also an effective agent for heavy metal removal from contaminated soil without further preparation. The costs for soil remediation using SLs can be reduced without the requirement for separation of SLs from the fermentation broth.

Considering the practical application and cost efficiency of sophorolipids for heavy metal removal, we compared the Cd and Pb removal efficiency by different types of sophorolipids produced from 1 L *S. bombicola* fermentation broth (Table 1). The total sophorolipids produced from 1 L fermentation broth in shaking flask was 57 g, which included 39 g acidic SLs and 18 g lactonic SLs. According to the above experimental results, addition of 100% (*v*/*v* %) fermentation broth, 1% (*w*/*v* %) crude lactonic SLs, 8% (*w*/*v* %) crude total SLs or acidic SLs resulted in the highest Cd and Pb removal efficiencies compared with other concentrations tested. For crude lactonic SLs, although 1 L fermentation broth could be prepared into 1.8 L 1% soil washing solution, the maximum metal removal by lactonic SLs was still the lowest because of its low heavy metal removal efficiency. Crude total SLs and acidic SLs extracted from 1 L fermentation broth could be prepared into 0.7125 L and 0.4875 L soil washing solution, respectively, at a concentration of 8%. Therefore, the amount of soil that can be treated by the two soil washing solutions was less than that treated by1 L directly used fermentation broth. In addition, the removal efficiency of Cd and Pb by undiluted fermentation broth was significantly higher than by 8% crude total SLs or acidic SLs solution. In general, 1 L fermentation broth without dilution (i.e., concentration 100%) could remove more Cd or Pb than other types of crude SL preparations. These results indicated that fermentation broth of *S. bombicola* used directly for heavy metal removal was a good candidate because of the low dosage required, high removal efficiency, and simple preparation.

### 3.4. FT-IR Study

**Figure 4:**
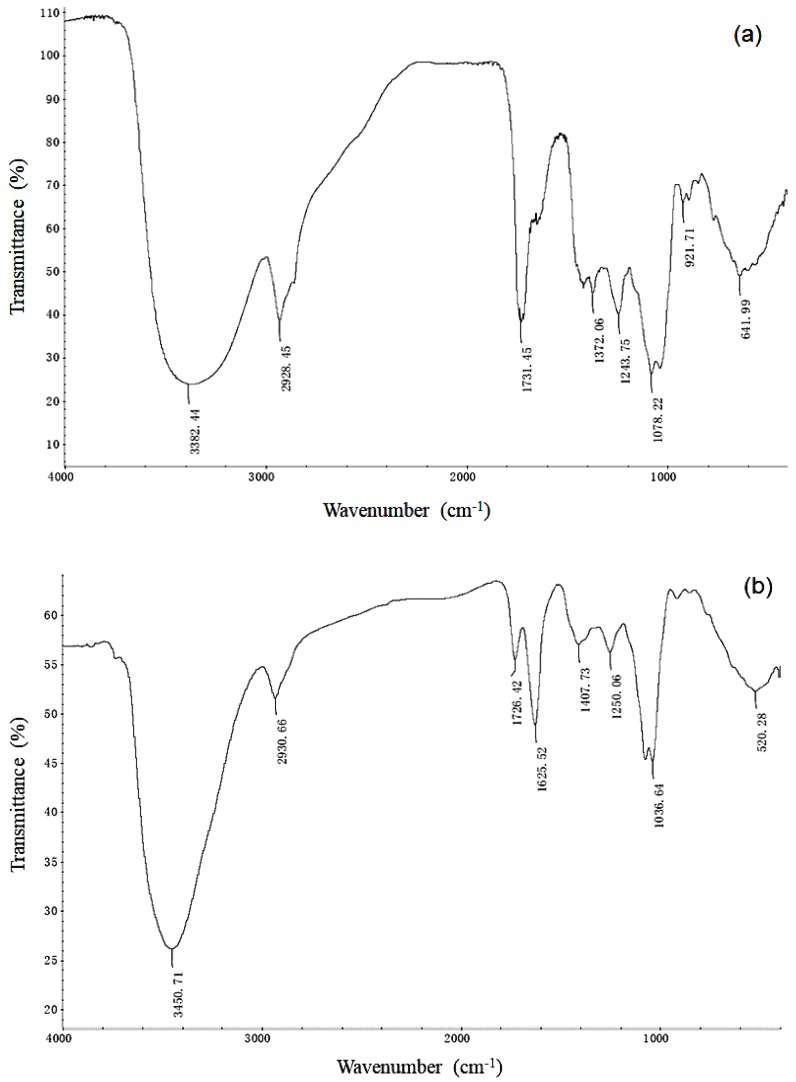
FT-IR spectroscopy of 8% crude acidic sophorolipids (**a**) and 8% crude acidic sophorolipids with Cd (**b**).

Acidic SLs fermented by *S. bombicola* were effective in removing heavy metals from soil according to the experimental results above. To investigate the interaction between Cd and crude acidic SLs, the Fourier-transform infrared analysis was performed. Figure 4a showed the FT-IR spectra of crude acidic SLs. The IR spectra of 3500–3200 and 2928 cm^−1^ revealed O-H stretch and CH_2_ groups, respectively. The C=O absorption band at 1731–1660 cm^−1^ may contain the contributions of esters or acids groups. The stretch of C-O band at 1243 cm^−1^ could be assigned to the acetyl ester, while sugar C-O stretch of C-O-H groups was found to be at 1078 cm^−1^ [25]. The FT-IR spectrum of crude acidic SLs with Cd was shown in Figure 4b. Compared to the results in Figure 4a, after Cd was added into crude acidic SLs solution, conspicuous changes in the FT-IR spectrum were the appearance of asymmetrical stretching and symmetrical stretching vibration band of O=C-O group at 1625 cm^−1^ and 1407 cm^−1^, respectively (Figure 4b). Furthermore, the band transmittance near 1726 cm^−1^ was significantly increased than that in Figure 4a.

FT-IR has been reported to be used to identify the changes of certain functional groups in a molecule after metal was added. Kim et al. [41] compared the FT-IR spectra of 10 CMC UH-biosurfactant (fermented by *Flavobacterium* sp.) and UH-biosurfactant with lead. They observed that the asymmetric stretching carboxyl group shifted (1706 to 1656 cm^−1^) due to the addition of lead into the UH-biosurfactant solution. The complexation of lead by the carboxyl group in the biosurfactant was effective in removing the heavy metal from the solution. Das et al. [42] also found a FT-IR spectral characteristic vibrational frequency shift after metal adsorbed to the crude biosurfactant which fermented by a marine bacterium. It was confirmed an ionic bonding between metal ions and the biosurfactant. In this study, the changes in FT-IR spectrum were similar to the results in the previous reports. The results indicated that the free carboxylic end of the fatty acid in acidic SLs formed complexes with Cd in the solution. The bonds of Cd—acidic SLs complexes were stronger than the bonds between Cd and the soil. The complexes desorpted from the soil into the washing solution and incorporated of the metal into biosurfactant micelle [43].

The possible mechanism for metal removal by the biosurfactants is that the complexes of surfactant with metal adsorb onto the soil surface, the metals are separated from the soil into the soil solution and hence associate with surfactant micelles. The anionic acidic SLs biosurfactant carrying negative charge formed an ionic bond with cationic metals, and the ionic bond is stronger than the association of the metals and the soil. The polar head groups of biosurfactant micelles can bind metals and make the metals more soluble in water [40,44]. Another probable reason is that the biosurfactant allows greater removal of heavy metals from soil due to its ability to decrease interfacial tension [15].

Mixed biosurfactants system containing nonionic lactonic SLs and anionic biosurfactant such as rhamnolipid solutions are expected to have superior efficiency in decreasing surface/interfacial tension and a lower CMC compared with individual component. The physical properties of mixtures depend on the ratio of nonionic to ionic surfactants [45]. Sophorolipids produced by *S. bombicola* is a mixture of anionic acidic and nonionic lactonic sophorolipids. The two different types of sophorolipids can be separately prepared and mixed by different proportions to treat different soils containing different heavy metals and organic contaminations. Therefore, the development of low-cost, simple, and environmentally friendly extraction methods of SLs is the direction of future research.

## 4. Conclusions

Biosurfactant sophorolipids produced by *Starmerella bombicola* CGMCC 1576 were able to remove Cd and Pb from contaminated soil. Cd and Pb removal were influenced by the types, pH, and concentrations of surfactants. The removal efficiencies of acidic and total SLs were better than distilled water and synthetic surfactants (SDS and Tween-80). Acidic SLs (anionic) were better than lactonic SLs (nonionic) in enhancing remediation of heavy metal-contaminated soils. The Cd and Pb removal efficiency increased with the increase in SLs concentrations. Low concentrations of SLs could remove more Cd than synthetic surfactants. When the concentration of SLs was above 4%, the removal of Pb was efficient. FT-IR analysis showed that the complexation of Cd by the carboxyl group in acidic SLs formed in the solution which was effective in removing Cd from the soil to the washing solution. The fermentation broth of *S. bombicola* without further preparation process can be directly used on Cd and Pb removal and the superior removal efficiency was reached with less dosage. This provides a potential bioremediation agent for heavy metal removal and makes it impossible to decrease costs of bioremediation for heavy metal-contaminated soil by biosurfactants.

## Figures and Tables

**Table 1:** Comparison of heavy metal removal efficiency by different types of sophorolipids produced from 1 L *S. bombicola* fermentation broth.

Metal	Biosurfactant ^1^	Biosurfactant Concentration	Maximum of Metal Removal (mg)
Cd	Fermentation broth	100%	3.38 ± 0.13
	Crude total SLs solution	8%	2.02 ± 0.04
	Crude acidic SLs solution	8%	1.45 ± 0.05
	Crude lactonic SLs solution	1%	0.65 ± 0.15
Pb	Fermentation broth	100%	3.45 ± 0.17
	Crude total SLs solution	8%	1.43 ± 0.07
	Crude acidic SLs solution	8%	1.45 ± 0.09
	Crude lactonic SLs solution	1%	0.29 ± 0.09

^1^ prepared from 1L *S. bombicola* fermentation broth.

## References

[1] Oves M., Khan M.S., Zaidi A., Ahmad E. (2012). Soil Contamination, Nutritive Value, and Human Health Risk Assessment of Heavy Metals: An Overview.

[2] EPA (The United States Environmental Protection Agency) (2004). Cleaning Up the Nation’s Waste Sites: Markets and Technology Trends.

[3] Luo X.S., Yu S., Zhu Y.G., Li X.D. (2012). Trace metal contamination in urban soils of China. Sci. Total Environ..

[4] Mulligan C.N. (2005). Environmental applications for biosurfactants. Environ. Pollut..

[5] Bolan N., Kunhikrishnan A., Thangarajan R., Kumpiene J., Park J., Makino T., Kirkham M.B., Scheckel K. (2014). Remediation of heavy metal(loid)s contaminated soils—To mobilize or to immobilize?. J. Hazard. Mater..

[6] EPA (The United States Environmental Protection Agency) (1997). Best Management Practices (BMPs) for Soils Treatment Technologies.

[7] Dermont G., Bergeron M., Mercier G., Richer-Lafleche M. (2008). Soil washing for metal removal: A review of physical/chemical technologies and field applications. J. Hazard. Mater..

[8] Mohamed M.A., Efligenir A., Husson J., Persello J., Fievet P., Fatin-Rouge N. (2013). Extraction of heavy metals from a contaminated soil by reusing chelating agent solutions. J. Environ. Chem. Eng..

[9] Torres L.G., Lopez R.B., Beltran M. (2012). Removal of As, Cd, Cu, Ni, Pb, and Zn from a highly contaminated industrial soil using surfactant enhanced soil washing. Phys. Chem. Earth Parts A/B/C.

[10] Ehsan S., Prasher S.O., Marshall W.D. (2006). A washing procedure to mobilize mixed contaminants from soil. J. Environ. Qual..

[11] Mulligan C.N., Yong R.N., Gibbs B.F. (1999). On the use of biosurfactants for the removal of heavy metals from oil-contaminated soil. Environ. Prog..

[12] Chen W.J., Hsiao L.C., Chen K.Y. (2008). Metal desorption from copper(ii)/nickel(ii)-spiked kaolin as a soil component using plant-derived saponin biosurfactant. Process Biochem..

[13] Franzetti A., Caredda P., Ruggeri C., Colla P.L., Tamburini E., Papacchini M., Bestetti G. (2009). Potential applications of surface active compounds by *Gordonia* sp. strain BS29 in soil remediation technologies. Chemosphere.

[14] Maity J.P., Huang Y.M., Fan C.W., Chen C.C., Li C.Y., Hsu C.M., Chang Y.F., Wu C.I., Chen C.Y., Jean J.S. (2013). Evaluation of remediation process with soapberry derived saponin for removal of heavy metals from contaminated soils in Hai-Pu, Taiwan. J. Environ. Sci..

[15] Juwarkar A.A., Nair A., Dubey K.V., Singh S.K., Devotta S. (2007). Biosurfactant technology for remediation of cadmium and lead contaminated soils. Chemosphere.

[16] Schippers C., Gessner K., Muller T., Scheper T. (2000). Microbial degradation of phenanthrene by addition of a sophorolipid mixture. J. Biotechnol..

[17] Kang S.W., Kim Y.B., Shin J.D., Kim E.K. (2010). Enhanced biodegradation of hydrocarbons in soil by microbial biosurfactant, sophorolipid. Appl. Biochem. Biotechnol..

[18] Qi X.Y., Song X., Sun Y.M. (2014). Enhanced soil washing of PCB by sophorolipids from transformer oil contaminated soil. Appl. Mech. Mater..

[19] Van Bogaert I.A., Soetaert W., Soberón-Chávez G. (2011). Sophorolipids. Biosurfactants.

[20] Fleurackers S.J.J. (2010). On the use of waste frying oil in the synthesis of sophorolipids. Eur. J. Lipid Sci. Technol..

[21] Mulligan C.N., Yong R.N., Gibbs B.F. (2001). Heavy metal removal from sediments by biosurfactants. J. Hazard. Mater..

[22] GB 15618-1995 (1995). Environmental Quality Standards for Soils.

[23] Li J., Li H., Li W., Xia C., Song X. (2016). Identification and characterization of a flavin-containing monooxygenase MoA and its function in a specific sophorolipid molecule metabolism in *Starmerella bombicola*. Appl. Microbiol. Biotechnol..

[24] Chen J., Song X., Zhang H., Qu Y. (2006). Production, structure elucidation and anticancer properties of sophorolipid from *Wickerhamiella domercqiae*. Enzyme Microb. Technol..

[25] Ma X.J., Li H., Shao L.J., Shen J., Song X. (2011). Effects of nitrogen sources on production and composition of sophorolipids by *Wickerhamiella domercqiae* var. *sophorolipid* CGMCC 1576. Appl. Microbiol. Biotechnol..

[26] EPA (The United States Environmental Protection Agency) (2007). Microwave Assisted Acid Digestion of Sediments, Sludges, Soils, and Oils.

[27] Hong K.J., Tokunaga S., Kajiuchi T. (2002). Evaluation of remediation process with plant-derived biosurfactant for recovery of heavy metals from contaminated soils. Chemosphere.

[28] Hong K.J., Choi Y.K., Tokunaga S., Ishigami Y., Kajiuchi T. (1998). Removal of cadmium and lead from soil using aescin as a biosurfactant. J. Surfactants Deterg..

[29] Serrano S., Garrido F., Campbell C.G., García-González M.T. (2005). Competitive sorption of cadmium and lead in acid soils of Central Spain. Geoderma.

[30] Doong R.A., Wu Y.W., Lei W.G. (1998). Surfactant enhanced remediation of cadmium contaminated soils. Water Sci. Technol..

[31] Torrens J.L., Herman D.C., Miller-Maier R.M. (1998). Biosurfactant (rhamnolipid) sorption and the impact on rhamnolipid-facilitated removal of cadmium from various soils under saturated flow conditions. Environ. Sci. Technol..

[32] Wang S., Mulligan C.N. (2009). Arsenic mobilization from mine tailings in the presence of a biosurfactant. Appl. Geochem..

[33] Gao L., Kano N., Sato Y., Li C., Zhang S., Imaizumi H. (2012). Behavior and distribution of heavy metals including rare earth elements, thorium, and uranium in sludge from industry water treatment plant and recovery method of metals by biosurfactants application. Bioinorg. Chem. Appl..

[34] Chang S., Wang K., Kuo C., Chang C., Chou C. (2005). Remediation of metal-contaminated soil by an integrated soil washing-electrolysis process. Soil Sediment Contam. Int. J..

[35] Miller R.M. (1995). Biosurfactant-facilitated remediation of metal-contaminated soils. Environ. Health Perspect..

[36] Herman D.C., Artiola J.F., Miller R.M. (1995). Removal of cadmium, lead, and zinc from soil by a rhamnolipid biosurfactant. Environ. Sci. Technol..

[37] Christofi N., Ivshina I.B. (2002). Microbial surfactants and their use in field studies of soil remediation. J. Appl. Microbiol..

[38] Aşçı Y., Nurbaş M., Açıkel Y.S. (2007). Sorption of Cd(ii) onto kaolin as a soil component and desorption of Cd(ii) from kaolin using rhamnolipid biosurfactant. J. Hazard. Mater..

[39] Singh P., Cameotra S.S. (2004). Enhancement of metal bioremediation by use of microbial surfactants. Biochem. Biophys. Res. Commun..

[40] Açıkel Y., Khan M.S., Zaidi A., Goel R., Musarrat J. (2011). Use of biosurfactants in the removal of heavy metal ions from soils. Biomanagement of Metal-Contaminated Soils.

[41] Kim J., Vipulanandan C. (2006). Removal of lead from contaminated water and clay soil using a biosurfactant. J. Environ. Eng..

[42] Das P., Mukherjee S., Sen R. (2009). Biosurfactant of marine origin exhibiting heavy metal remediation properties. Bioresour. Technol..

[43] Liu G., Zhong H., Yang X., Liu Y., Shao B., Liu Z. (2018). Advances in applications of rhamnolipids biosurfactant in environmental remediation: A review. Biotechnol. Bioeng..

[44] Mulligan C.N. (2009). Recent advances in the environmental applications of biosurfactants. Curr. Opin. Colloid Interface Sci..

[45] Song D., Li Y., Liang S., Wang J. (2013). Micelle behaviors of sophorolipid/rhamnolipid binary mixed biosurfactant systems. Colloids Surf. A Physicochem. Eng. Aspects.

